# Open Biology's first few months

**DOI:** 10.1098/rsob.120027

**Published:** 2012-02

**Authors:** David Glover

## Meeting the needs of researchers

When we launched *Open Biology* late last year, our goal was to offer a high-quality, open access journal that met the needs of scientific researchers working in biology at the molecular and cellular level. I feel that we are well on our way to achieving this goal and wish to update you on the progress we have made so far.

*Open Biology*'s mission is to provide a service that actively meets the needs of biologists with cellular and molecular interests. This includes providing a rapid, constructive and fair peer review system that allows valuable work to proceed quickly to publication. At the launch event, the President of the Royal Society, Paul Nurse, succinctly summarized the essence of our ethos:

This new journal will be run by scientists for scientists with an editorial system that will be making decisions rather than weighing opinion.

I have been extremely encouraged by the feedback I have received from our authors so far, who have commented on the speedy turnaround, excellent reviews and decisive handling of the papers by Editors who provide constructive, scientific input.

## Open biology so far …

The challenge with a new journal is to build and maintain momentum. Since launching, we have published a relatively small number of articles. However, I am very pleased with the quality and potential impact of the articles published so far. In these first few months, we have published papers that cover such diverse topics as the structural biology of the transketolase from *Mycobacterium tuberculosis*, a novel drug target for the treatment of tuberculosis [1], to the mathematical modelling of START, the point at which a yeast cell becomes committed to undertake a new cell division cycle [2].

Of particular interest is an elegant report from Mariann Bienz' laboratory that uses *Drosophila* as a model to study how the *Adenomatous polyposis coli* (APC) tumour suppressor enables Axin to promote the degradation of the Wnt signalling effector β-catenin [3]. By examining *apc* null mutant *Drosophila* tissues, they discovered parallels with *APC* mutant human tumour cells in attenuating Axin degradasomes assembly. Their results suggest that APC both promotes Axin to assemble into degradosomes and also opposes its inactivating interaction with Dishevelled.

Dario Alessi and Miratul Muqit at the MRC Protein Phosphorylation Unit in Dundee sent us their study on the mutation of PINK1 kinase in Parkinson's disease [4]. This field has been plagued by the low activity of the human enzyme. To get around this, they have developed a system to use the insect counterparts of PINK1 kinase to examine the consequences of known disease-associated mutations in the enzyme.

We also attracted a very exciting report from Mitsuhiro Yanagida's group about condensin, a chromosome-associated protein whose diverse roles are poorly understood. Their study suggests that condensin can unwind DNA to allow proteins such as the replication protein A to be removed from chromosomes after DNA repair and before the onset of mitosis [5].

Another fascinating study came from Neil Barclay and colleagues who examined the stability of a disulphide bond in CD132, part of the receptor for the cytokines, interleukins-2 and -4 [6]. Enzymes secreted during immune activation can reduce this bond leading to inhibition of STAT-5 signalling and of proliferation of a T-cell clone. Interestingly, mutants in these residues lead to immunodeficiency in humans.

I am grateful to these and all of our authors for sending the results of their labours to us. I am also indebted to the work of the Subject Editors, Editorial Board and Royal Society Editorial Office in handling submission, peer review and publication in a timely manner. One priority is to get *Open Biology* indexed in the appropriate databases, the most important of which is *PubMed*. In order for *PubMed* to evaluate a new journal, they need a minimum number of published articles and I am pleased to report that we are to make our submission for inclusion very soon. In addition, I can confirm that we are already being tracked for inclusion in *Web of Science* and *Journal Citation Reports*.

## Open access

By launching *Open Biology*, the Society continues to demonstrate its support for open access publishing. The journal will be funded by charges for articles accepted for publication. Since launch, we have offered a promotional waiver for these charges. However, in order to make publication sustainable, these charges will apply for accepted papers submitted from 1 March. I would like to take this opportunity to explain a little about how this works.

The Society has set a price of £1200 for the article processing charge. This charge covers the costs of peer review, composition, hosting and archiving. While the charge is slightly higher than some other open access journals, it is linked to the fact that *Open Biology* is a selective journal with a relatively high rejection rate. Therefore, this price reflects the fact that we are accepting (and therefore charging for) a lower proportion of articles than less-selective journals.

Another means of funding open access publication is institutional open access membership. Such memberships allow institutions to pay an annual fee and in return researchers at the institution receive a discount to the article processing charge. The Royal Society has recently launched membership programmes for all its journals, including *Open Biology.* It has received a positive reception and a number of key institutions have already signed up. I am encouraged by this move by institutions to support open access and benefit their authors by lowering the financial barrier for publication.

## Moving forward

It is still very early days for *Open Biology*, but I am encouraged by its reception in the scientific community and I welcome your feedback. The Royal Society has a long record of scientific publishing, originating with its oldest journal, *Philosophical Transactions*, which spans three and a half centuries (http://trailblazing.royalsociety.org/). I feel confident that we can add to this rich history in this very latest forum for communication between scientists. I would like to encourage all of my fellow biologists to join with us in making our newest journal an equal success.

## References

[1] Fullam E Pojer F Bergfors T Jones TA Cole ST 2012 Structure and function of the transketolase from *Mycobacterium tuberculosis* and comparison with the human enzyme. Open Biol. 2, 110026 10.1098/rsob.110026 (doi:10.1098/rsob.110026) 22645655 PMC3352088

[2] Zhang T Schmierer B Novák B 2011 Cell cycle commitment in budding yeast emerges from the cooperation of multiple bistable switches. Open Biol. 1, 110009 10.1098/rsob.110009 (doi:10.1098/rsob.110009) 22645649 PMC3352082

[3] Mendoza-Topaz C Mieszczanek J Bienz M 2011 The *Adenomatous polyposis coli* tumour suppressor is essential for Axin complex assembly and function and opposes Axin's interaction with Dishevelled. Open Biol. 1, 110013 10.1098/rsob.110013 (doi:10.1098/rsob.110013) 22645652 PMC3352083

[4] Woodroof HI 2011 Discovery of catalytically active orthologues of the Parkinson's disease kinase PINK1: analysis of substrate specificity and impact of mutations. Open Biol. 1, 110012 10.1098/rsob.110012 (doi:10.1098/rsob.110012) 22645651 PMC3352081

[5] Akai Y 2011 Opposing role of condensin hinge against replication protein A in mitosis and interphase through promoting DNA annealing. Open Biol. 1, 110023 10.1098/rsob.110023 (doi:10.1098/rsob.110023) 22645654 PMC3352087

[6] Metcalfe C Cresswell P Barclay AN 2012 Interleukin-2 signalling is modulated by a labile disulfide bond in the CD132 chain of its receptor. Open Biol. 2, 110036 10.1098/rsob.110036 (doi:10.1098/rsob.110036) 22645657 PMC3352089

